# Terrestrial mosses as a substrate and potential host for cyanobacteria, green algae and diatoms

**DOI:** 10.1007/s10265-024-01551-2

**Published:** 2024-06-27

**Authors:** Ewelina Szczepocka, Paulina Nowicka-Krawczyk, Grzegorz J. Wolski

**Affiliations:** 1 Department of Algology and Mycology, Faculty of Biology and Environmental Protection, University of Lodz, Banacha 12/16, Lodz, 90-237 Poland; 2 Department of Geobotany and Plant Ecology, Faculty of Biology and Environmental Protection, University of Lodz, Banacha 12/16, Lodz, 90-237 Poland

**Keywords:** Epiphytic organisms, Humidity, Micromorphology of gametophyte leaves, Temperate climate zone, Terrestrial bryophytes

## Abstract

Most studies of terrestrial bryophytes as natural substrates for photosynthetic microorganisms have been performed in the polar regions, where bryophytes are an important part of the ecosystem. As they remain green throughout the year, bryophytes may also be an ideal substrate for epiphytic organisms in temperate regions. The present study investigated the colonization potential and diversity of microalgae on selected plant species in riparian forest and spruce monoculture in a temperate region. It examines whether the presence of algae is related to substrate humidity, the micromorphology of gametophyte or the seasonal availability of substrate. The taxonomic diversity of algae was studied. Cyanobacteria and green algae were cultured on BG-11 agar medium, while diatoms were identified in permanent diatomaceous slides. The alpha- and beta-diversity indices were calculated, and the communities were compared using Bray-Curtis distances and multidimensional correspondence analyses. Our findings indicate that the largest number of alga species were diatoms; however, their presence was only observed in riparian forest and was associated with high humidity. Both aerophilic and freshwater taxa were noted, the latter carried by water from nearby aquatic ecosystem. Green algae were present in both phytocoenoses and humidity appears to have no substantial effect on the degree of colonization; their diversity was low and the group consisted of terrestrial taxa. In two bryophytes growing at the highest humidity, cyanobacteria were only identified in culture. The key factor influencing the degree of microalgae colonization was the humidity of the substrate, which was related to the distance from water.

**Supplementary Information:**

The online version contains supplementary material available at 10.1007/s10265-024-01551-2.

## Introduction

Terrestrial microalgae are able to colonize a range of natural and anthropogenic substrates (Sharma et al. 2007), particularly the walls of buildings, rocks, tree bark or soil. However, in the terrestrial environment, they experience intense radiation, fluctuations in humidity as far as complete drying, and high temperature ranges, from freezing to over 50 °C in some cases. In response, successful terrestrial algae have evolved various complex biochemical and physiological adaptations. Common adaptations include changes in the proportion of photosynthetic pigments, the production of slimy sugar-lipid cell envelopes, and cryo- and UV-radiation protective compounds (Holzinger and Karsten 2013; Nowicka-Krawczyk et al. 2022); moreover, some phototrophs may even switch to heterotrophy under UV-stress conditions (Gustavs et al. 2016).

Algae grow as epiphytes of higher plants and terrestrial bryophytes, and the latter is able to provide a unique habitat for algae: the dense bryophyte turf protects algal cells against intense radiation and violent weather events (Celewicz-Gołdyn and Kuczyńska-Kippen 2017). So far, research on the relationship between these groups of organisms has been conducted mainly in polar ecosystems, whose phytocoenoses are dominated by bryophytes and lichens. However, this research is generally concerned with the relationship between the level of substrate moisture and the composition of the algal species inhabiting the substrates (Bishop et al. 2021; Van de Vijver and Beyens 1999; Van de Vijver et al. 2008). Some studies have also examined the relationship between the structure of the gametophyte, its exposure to the sun and the retention of moisture in the turf of bryophytes occurring on exposed cliff walls (Ress and Lowe 2014). However, few such reports have been carried out in temperate areas. Some studies have examined terrestrial algae colonizing bryophytes growing on the damp walls of buildings or damp bark of trees; their findings show that the organisms constitute a substrate which provides favourable conditions for the development of algae due to the possibility of collecting water and organic substances (Nowicka-Krawczyk et al. 2014; Rybak et al. 2018).

Since microalgae are poikilohydric organisms, bryophytes, especially their gametophytes, easily retain and maintain their moisture (Schofield 1985), and may be important microhabitats for the development of epiphytic algae in temperate climate zones. The present study examines the colonization potential of microalgae with regard to their distance from aquatic ecosystems. Its main objective was to investigate the taxonomic diversity of algae growing on selected terrestrial moss species occurring in two different forest phytocoenoses: riparian forest and spruce monoculture. The following aims were set:

i. Determining which of the studied plant species is characterized by the highest diversity of microalgae;
ii. Determining the effect of substrate moisture on algae community structure in individual substrates;
iii. Confirming whether the micromorphology of moss leaves affects the degree of colonization by microalgae;
iv. Confirming whether the presence of microalgae is related to the seasonal stability of the tested substrate.

## Materials and methods

### Study area

**Fig. 1:**
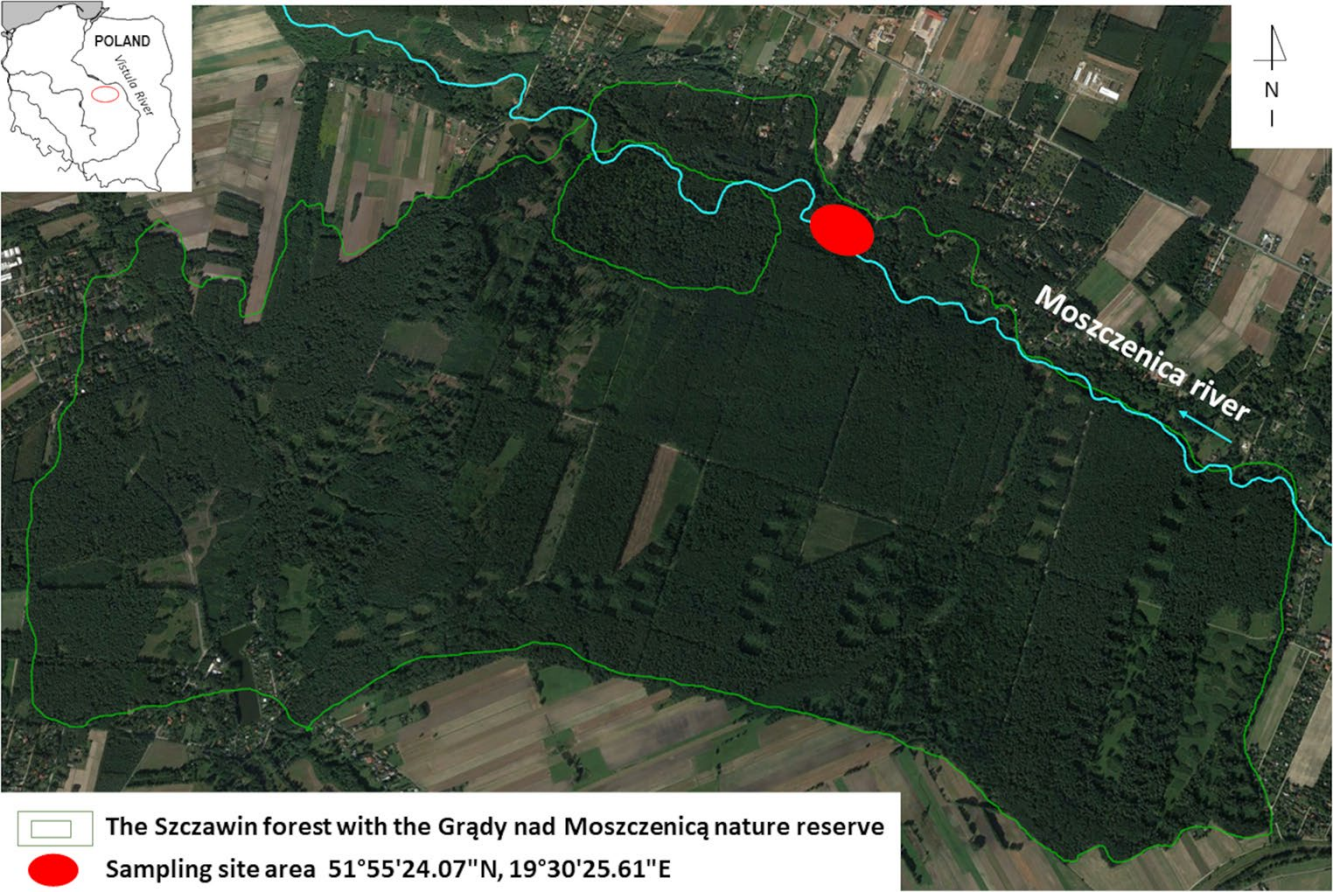
Location of the sampling area

The research was carried out in the northern part of the Szczawin Forest District, Zgierz, in the vicinity of the Grądy nad Moszczenica nature reserve, Lodz Voivodeship, Poland (Fig. 1). The Moszczenica river passes the Szczawin forest at a distance of 17 km from its springs, meandering in a valley, with a depth of up to 2–3 m. The sampling area was verified taking into consideration the distance from the river and the shape of the terrain.

The material was collected in the early spring of 2022, of which, six mosses and one vascular plant, *Ficaria verna* Huds., were taken for study. The latter has a short vegetation period, and hence was selected as a background to check whether the presence of microalgae is related to the seasonal availability of the host plant; the plant itself has only been marginally studied to date. *Ficaria verna* was collected from soil in riparian forests where it grew close to the Moszczenica riverbank (ca. 30 cm), in an area periodically flooded by water. At the same distance, on slightly-elevated ground above the river (ca. 1 m), one epigeic (terricolous) moss, *Plagiothecium longisetum* Lindb., was collected. Two other moss samples, *viz. Plagiomnium undulatum* (Hedw.) T.J. Kop. and *Atrichum undulatum* (Hedw.) P. Beauv., were collected from soil in the riparian forest, at a location between four and six meters from the river, where the ground was slightly elevated, and the soil was waterlogged. Although this area did not have any direct connection to the river flow, freshwater may affect moss turfs after heavy rains. Additionally, a species of epiphytic moss, *Hypnum cupressiforme* Hedw., was also collected at a height of 50 cm from the bark of two trees, *Carpinus betulus* L. and *Acer pseudoplatanus* L.; the location was 20 m from the river in the riparian phytocoenosis area. Finally, two terricolous mosses, *Polytrichum formosum* Hedw. and *Dicranum polysetum* Sw. *ex* anon, were collected; these were obtained farthest from the river site, in the spruce monoculture area. The moss species were also chosen due to their differences in leaf morphology: while most have smooth leaves without any lamellae, *A. undulatum* has a few lamellae and *P. formosum* has a high number.

Five subsamples of moss-algal material were collected from each of the studied plants, i.e. 40 subsamples in total, for phycological analysis. In addition, the relative humidity (% Rh) of the moss turf and macrophyte clumps was investigated using a Testo 606-2 thermohygrometer (Testo, Warsaw, Poland); five replicates were taken for each turf (Table 1).

**Table 1:** The relative humidity (% Rh), given as range and mean value, detected on moss turfs and macrophyte clump; air temperature t = 14.7 °C

	The spruce monoculture – terricolous species	The riparian forest – terricolous species	The riparian forest -epiphytes on tree
*Polytrichum formosum*	11.3 ÷ 12.1 (11.6)		
*Dicranum polysetum*	11.1 ÷ 11.7 (11.3)		
*Plagiothecium longisetum*		24.8 ÷ 25.9 (25.2)	
*Ficaria verna*		20.3 ÷ 22.4 (21.1)	
*Plagiomnium undulatum*		20.0 ÷ 20.7 (20.3)	
*Atrichum undulatum*		20.3 ÷ 20.8 (20.5)	
*Hypnum cupressiforme* (on *Carpinus betulus*)			0.2 ÷ 1.5 (0.8)
*Hypnum cupressiforme* (on *Acer pseudoplatanus*)			0.5 ÷ 1.3 (0.9)

### Qualitative and quantitative analysis of algal assemblages

Due to methodological differences, the cyanobacteria and green algae were investigated separately to the diatoms, and the algae were subjected to two different methods of taxonomical analysis.

To investigate the first group of algae, phycological samples were carefully taken from the leafy stems of bryophytes and leaf parts of the host macrophyte into 5 ml of BG-11 liquid medium (Andersen 2005), using one sterile soft brush for each subsample. All subsamples for each host species were pooled, and 1 ml of the algal suspension was transferred into four 1.5% BG-11 agar plates and incubated for one month under the following conditions: artificial light (2800 lx) from fluorescent tubes (Osram FLUORA T8 L 36 W/77) in a 16 h/8 h day/night period, temperature of 22 °C ± 0.2 °C and air humidity of 50% ± 5%. The remaining sample was subjected to microscope examination immediately after inoculation to check for the presence of any uncultured taxa.

The initial and cultivated samples were subjected to algal taxonomic analysis using a Nikon Eclipse 50i Light Microscope with DIC optics (Precoptic Co., Warsaw, Poland) and Opta-Tech documentation system. All investigations were performed at 10 × 40 and 10 × 60 magnifications, and the identification was made according to Komárek and Anagnostidis (1999; 2007), Bock et al. (2011), Komárek (2013), Darienko et al. (2015), Mikhailyuk et al. (2015), Pröschold and Darienko (2020), John et al. (2021). Since all taxa from the initial observation were also present on the agar plates, the total number of colonies of a particular taxon on four replicates of agar plates was counted and used for quantitative assessment.

The diatoms colonizing the selected mosses and macrophyte were identified using solid preparations. Fragments of the collected plants were placed in 12 ml test tubes and suspended in a mixture of sulphur and chrome acid; this procedure not only lyses plant tissues but also destroys the diatom cell protoplast, leaving only the silica cell walls: the key diacritical feature for diatom taxonomic analysis (Round et al. 1990). The resulting diatomaceous material was embedded in Naphrax resin (Brunel Microscopes Ltd UK & International Specialists) creating permanent diatomaceous slides (Pliński and Witkowski 2009). These were then examined using a Nikon Eclipse 50i microscope (Precoptic Co., Warsaw, Poland) with an Opta-Tech documentation system at 10 × 40 and 10 × 100 magnifications. The qualitative analysis was performed according to Hofmann et al. (2011) and Lange-Bertalot et al. (2017), while the quantitative analysis was carried out based on around 400 valves in each permanent slide (Cholnoky 1968). To correctly interpret the occurrence of the diatoms, their species preference regarding the type of environment (moisture/aerophilic) according to Van Dam et al. was checked using Omnidia 6.1 (Lecointe et al. 1993), with the newly-related database (released on the 25th of March 2023).

### Mathematical analysis

To assess the biodiversity of the tested substrates (i.e., the plants overgrown by algae), alpha-diversity indices (Dominance D and Shannon H) were calculated for the green algal and diatom communities separately. The differences between the substrates were then evaluated using the permutation method (*p* ≤ 0.05), and the obtained p-values were subjected to the Benjamini-Hochberg correction for multiple comparisons (Benjamini and Hochberg 1995).

In addition, the Whittaker beta-diversity index was calculated, and to determine the similarity between the substrates, Bray-Curtis distances were calculated based on the frequency of occurrence of individual algal taxa. The grouping of individual substrates (viz. the mosses and the vascular plant) was then analysed in terms of the presence of algae on them: briefly, a correspondence analysis (CA) was performed, and then the obtained coordinates (Euclidean distance, Ward link method) were used to group the points corresponding to individual species and substrates. The calculations were performed using the PAST 4.09 package software (University of Oslo, Norway).

Following this, multivariate data analysis was performed to visualize the qualitative and quantitative differences in algal community structure between individual host plants, as explained by environmental data. The analysis was performed using Canoco 5.1. software (Microcomputer Power, New York, USA). Since the response data were compositional, with a gradient of 4.6 SD, a unimodal method was selected: Canonical Correspondence Analysis (CCA). All data were logarithmically transformed and standardized, and the Shannon-Wiener diversity index was calculated to allow comparison.

## Results

In total, 100 taxa of terrestrial algae, viz. cyanobacteria, green algae and diatoms, were recorded on seven plant species. The least numerous group were the Cyanobacteria, which were visible only in the cell cultures established on BG-11 agar plates. The green algae were represented by seven taxa, including two species of the *Klebsormidium* genus, *K*. *flaccidum* and *K*. *subtile*. The most numerous group were the diatoms, of which 90 taxa were recorded. The two genera *Navicula* and *Nitzschia* were represented by the largest number of species, ten and nine taxa, respectively, with as many as 20 genera being represented by only one species (Table S1).

**Fig. 2:**
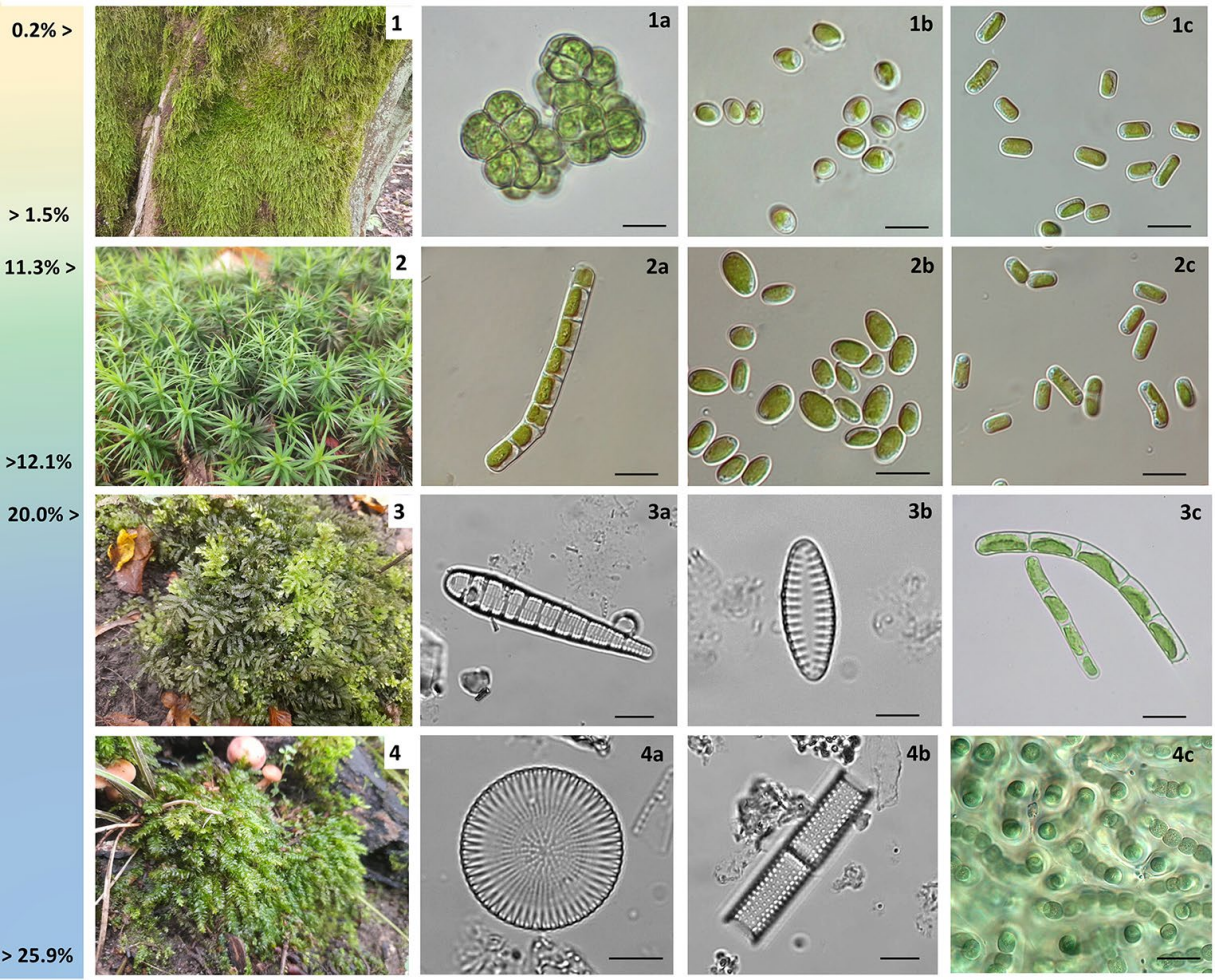
Selected bryophyte hosts and their frequent algal colonizers identified in the study, given as increasing humidity of turfs. **1**. *Hypnum cupressiforme*: **1a**. *Desmococcus olivaceus*, scale bar 20 μm, **1b**. *Pseudochlorella signiensis*, scale bar 10 μm, **1c**. *Pseudostichococcus* cf. *monallantoides*, scale bar 5 μm; **2**. *Polytrichum formosum*: **2a**. *Klebsormidim subtile*, scale bar 10 μm, **2b**. *Pseudochlorella signiensis*, scale bar 10 μm, **2c**. *Pseudostichococcus* cf. *monallantoides*, scale bar 5 μm; **3**. *Plagiomnium undulatum*: **3a**. *Meridion circulare*, scale bar 5 μm, **3b**. *Staurosira pinnata*, scale bar 5 μm, **3c**. *Klebsormidium flaccidum*, scale bar 10 μm, **4**. *Plagiothecium longisetum*: **4a**. *Cyclostephanos* cf. *dubius*, scale bar 10 μm, **4b**. *Aulacoseira granulata*, scale bar 10 μm, **4c**. *Desmonostoc* cf. *muscorum*, scale bar 10 μm

Photographic documentation of selected species of bryophytes and their frequent algal colonizers is presented in Fig. 2.

### Cyanobacteria

Only three taxa of blue-green algae were recorded, two of which (*Desmonostoc* cf. *muscorum*, *Gloeocapsa* sp.) were observed on a *Plagiothecium longisetum* growing on a slope in a riparian forest, whereas *Microcoleus terrestris* was recorded on *Ficaria verna*, also in the riparian forest. These were the only species on which any cyanobacteria were recorded: they were not recorded on bryophytes growing on the soil in the spruce monoculture (*Polytrichum formosum*, *Dicranum polysetum*), nor on *Hypnum cupressiforme* growing on the bark of *Carpinus betulus* or *Acer pseudoplatanus*, nor on other riparian forest taxa (*Plagiomnium undulatum*, *Atrichum undulatum*). *Microcoleus terrestris* was recorded twice, while the remaining taxa of cyanobacteria were recorded once (Table S1).

### Green algae

During the research, seven taxa of green algae were recorded. They were noted on all plant species: five taxa were noted on *Polytrichum formosum, Dicranum polysetum*, *Atrichum undulatum*, and *Hypnum cupresiforme* on *Acer pseudoplatanu*s, while four taxa were found on *Plagiothecium longisetum*, *Ficaria verna*, *Plagiomnium undulatum* and *Hypnum cupressiforme* (on *Carpinus betulus*). During the research, *Pseudochlorella signiensis* and *Pseudostichococcus* cf. *monallantoides* were most often noted (up to 42 and 33 records, respectively), and *Klebsormidium subtile* and *Coccomyxa* sp. the least (up to seven records and a single, respectively) (Table S1).

Similarity analysis (calculated by the Bray-Curtis measure) found the following pairs of plants to demonstrate the most similar compositions of green algae: *Hypnum cupressiforme* (on *Carpinus betulus*) and *Hypnum cupressiforme* (on *Acer pseudoplatanus*) (0.939); *Ficaria verna* and *Plagiomnium undulatum* (0.92); *Polytrichum formosum* and *Dicranum polysetum* (0.808); *Ficaria verna* and *Atrichum undulatum* (0.75). While the least similar were *Dicranum polysetum*, *Ficaria verna* and *Plagiomnium undulatum* (0.041); *Polytrichum formosum*, *Ficaria verna* and *Plagiomnium undulatum* (0.043) (Table S2).

Significant differences (*p* ≤ 0.05) were found between most plants with regard to the investigated alpha-diversity indices (Dominance D and Shannon H). Of the 28 pairs examined, only 11 (e.g., *Polytrichum formosum* and *Dicranum polysetum*; *Polytrichum formosum* and *Plagiothecium longisetum*; *Polytrichum formosum* and *Atrichum undulatum*) did not demonstrate any such significant differences. However, significant differences were observed for the other 17 pairs (e.g., *Polytrichum formosum* and *Ficaria verna*; *Polytrichum formosum* and *Plagiomnium undulatum*, *Polytrichum formosum* and *Hypnum cupressiforme*) (Table S3).

Regarding the beta-diversity, analysed by the Whittaker measure, the greatest diversity in recorded green algae was found between the following pairs: *Polytrichum formosum* and *Plagiothecium longisetum*; *Polytrichum formosum* and *Ficaria verna*; *Polytrichum formosum* and *Plagiomnium undulatum*; *Dicranum polysetum* and *Plagiothecium longisetum*; *Dicranum polysetum* and *Ficaria verna*; *Dicranum polysetum* and *Plagiomnium undulatum*; *Hypnum cupressiforme* and *Plagiothecium longisetum*; *Hypnum cupressiforme* and *Ficaria verna*; *Hypnum cupressiforme* and *Ficaria verna*. The remaining pairs of species are characterized by lower differences in biodiversity (Table S4).

**Fig. 3:**
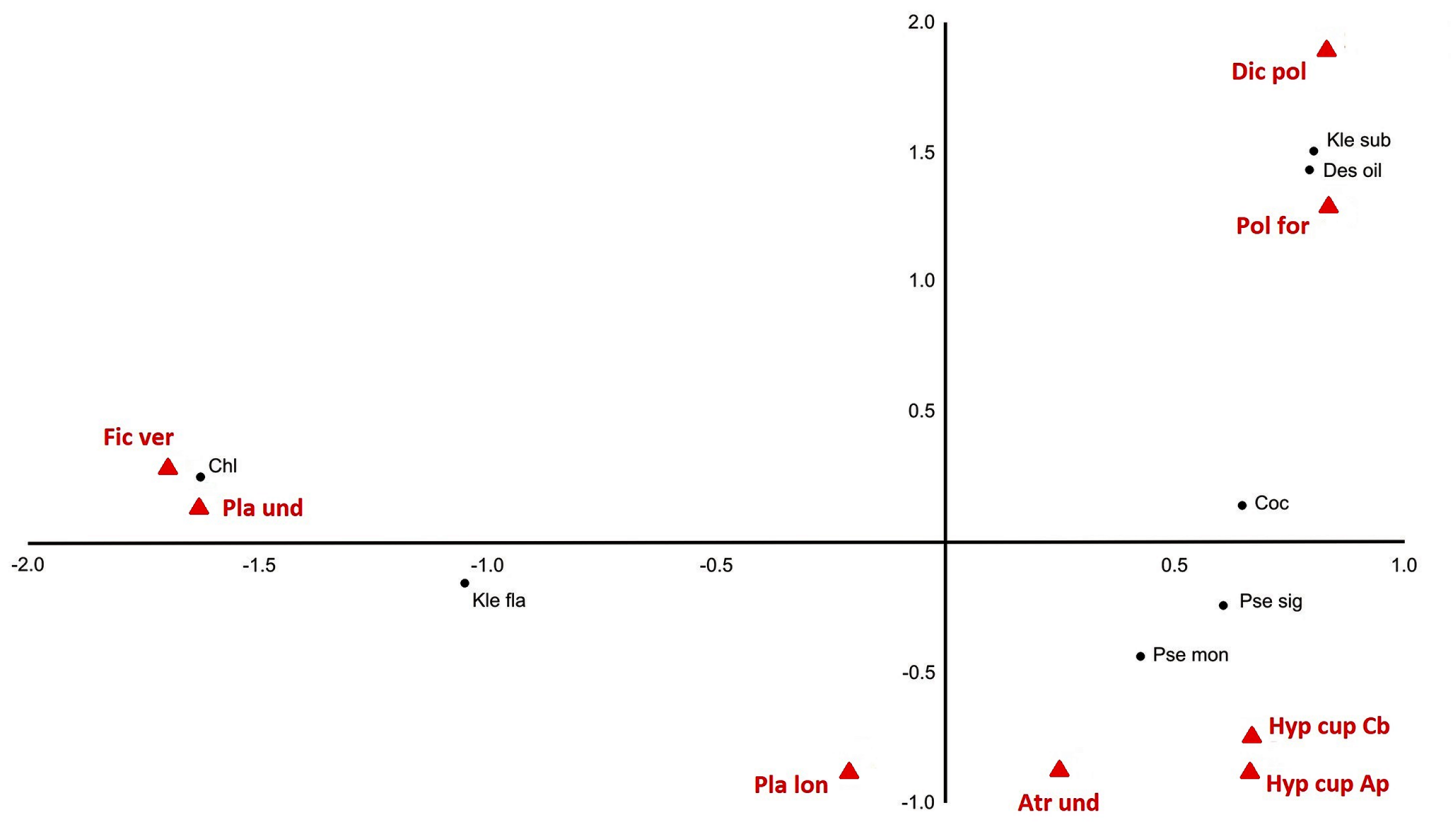
Correspondence analysis of green algae growing on particular plant species. Pol for – *Polytrichum formosum*; Dic pol – *Dicranum polysetum*; Pla lon – *Plagiothecium longisetum*; Fic ver – *Ficaria verna*; Pla und – *Plagiomnium undulatum*; Atr und – *Atrichum undulatum*; Hyp cup Cb *– Hypnum cupressiforme* (on *Carpinus betulus*); Hyp cup Ap – *Hypnum cupressiforme* (on *Acer pseudoplatanus*), acronyms for green algae are in Table S1

The CA showed clear groupings of green algae recorded on tested plants. *Polytrichum formosum* and *Dicranum polysetum* growing in spruce monoculture are grouped with *Klebsormidium subtile* and *Desmococcus olivaceus*; *Atrichum undulatum*, *Plagiothecium longisetum*, *Hypnum cupressiforme* from the riparian forest are associated with *Pseudostichococcus* cf. *monallantoides* and *Pseudochlorella signiensis*. In addition, *Ficaria verna* and *Plagiomnium undulatum* from the riparian forest form a group with *Chlorella* sp. and *Klebsormidium flaccidum* (Fig. 3). The analysis explains 89% of the data variability (axis X explains 64% variability, while Y 25%).

**Fig. 4:**
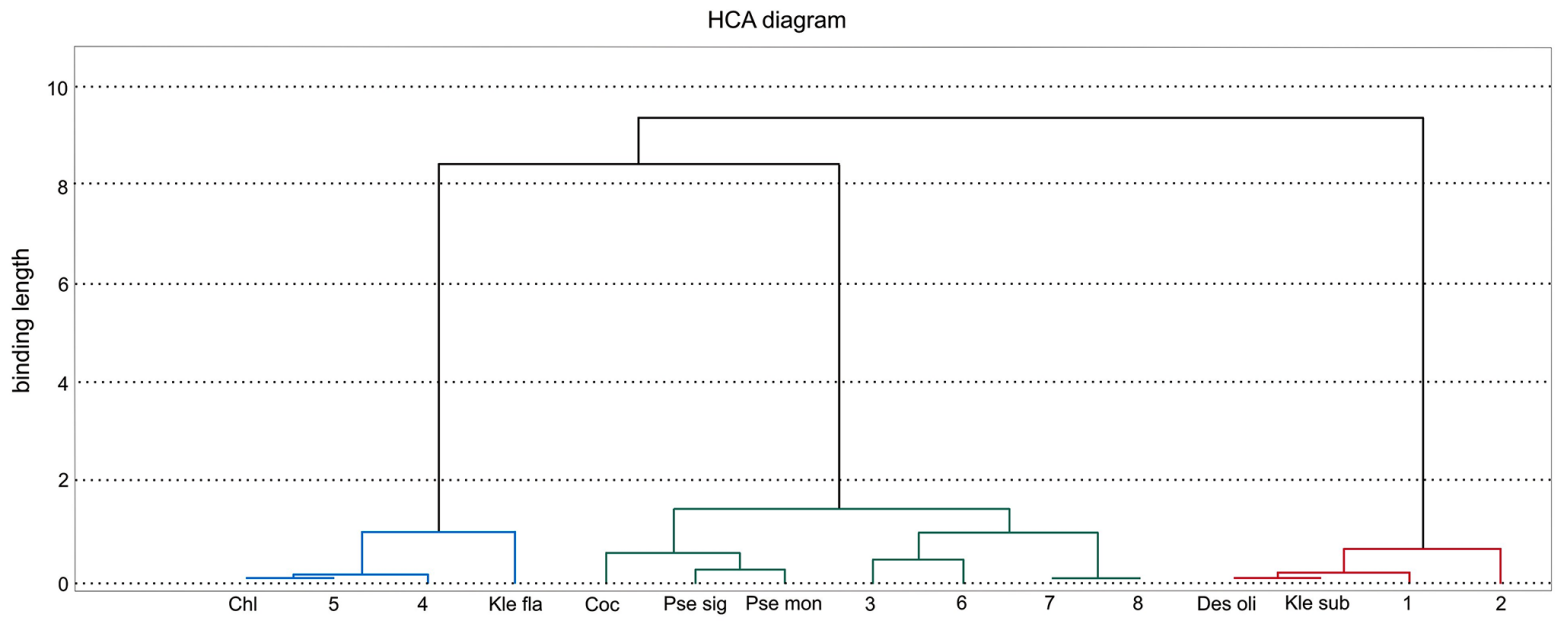
Dendrogram of similarity (Euclidean distance, Ward method) between plants and green algal colonizers. Explanation: 1 – *Polytrichum formosum*; 2 – *Dicranum polysetum*; 3 – *Plagiothecium longisetum*; 4 – *Ficaria verna*; 5 – *Plagiomnium undulatum*; 6 – *Atrichum undulatum*; 7 – *Hypnum cupressiforme* (on *Carpinus betulus*); 8 – *Hypnum cupressiforme* (on *Acer pseudoplatanus*), acronyms for green algae are in Table S1

These groupings are in line with the similarity dendrogram and confirm the presence of the three groups described above. One group comprises the mosses *Polytrichum formosum* and *Dicranum polysetum*, and their associated green algae, recorded on soil in a spruce monoculture. Another group comprises three species of mosses and their related green algae: two terricolous riparian species, *viz. Atrichum undulatum* and *Plagiothecium longisetum*, and the epiphyte *Hypnum cupressiforme* growing on *Carpinus betulus* and *Acer pseudoplatanus*. The third group comprises the riparian forest species *Ficaria verna* and *Plagiomnium undulatum* (Fig. 4).

### Diatoms

During the research, 90 diatom taxa were recorded; however, they were observed on only four plant species: three mosses and a vascular plant. No diatoms were recorded on *Polytrichum formosum*, *Dicranum polysetum* (terricolous species from a spruce monoculture) or *Hypnum cupressiforme* (noted in the riparian forest on the bark of *Acer pseudoplatanus* and *Carpinus betulus*). Diatoms were more commonly observed on other plant species, with the largest number on *Plagiothecium longisetum* (58 taxa), followed by *Ficaria verna* (45 taxa), by *Plagiomnium undulatum* (36 taxa) and *Atrichum undulatum* (19 taxa) (Table S1). The most commonly-observed diatoms were *Cyclostephanos* cf. *dubius* (120 records), *Staurosirella pinnata* (98 records) and *Achnanthidium minutissimum* (75 records); however, 21 taxa were found with only a single occurrence, among these were *Caloneis amphisbaena*, *Diatoma vulgaris Eucocconeis alpestris*, *Cymatopleura solea*, and *Diadesmis confervacea* (Table S1).

Similarity analysis, calculated using the Bray-Curtis measure, indicated that *Plagiothecium longisetum* and *Ficaria verna* demonstrated the most similar diatom species compositions (0.607). In contrast, the least similar pairs were found to be *Ficaria verna* and *Atrichum undulatum* (0.113) and *Plagiothecium longisetum* and *Atrichum undulatum* (0.131) (Table S5). Three pairs of mosses were found to demonstrate significant differences (*p* ≤ 0.05) in the investigated alpha-diversity indicators (Dominance D and Shannon H): *Plagiothecium longisetum* and *Ficaria verna*; *P*. *longisetum* and *Plagiomnium undulatum* (*p* < 0.01); *P*. *longisetum* and *Atrichum undulatum* (*p* = 0.01). No significant differences were noted for the three remaining pairs (Table S6).

Regarding the biodiversity of the overgrown substrate (i.e., beta-diversity analysed with the Whittaker measure), the greatest variation in recorded diatom composition was found between *Ficaria verna* and *Atrichum undulatum* (0.75) and between *Plagiothecium longisetum* and *A*. *undulatum* (0.66). In contrast, the least variation in biodiversity was found between *P*. *longisetum* and *F*. *verna* (0.44) and between *Plagiomnium undulatum* and *P*. *longisetum* (0.57) (Table S7).

**Fig. 5:**
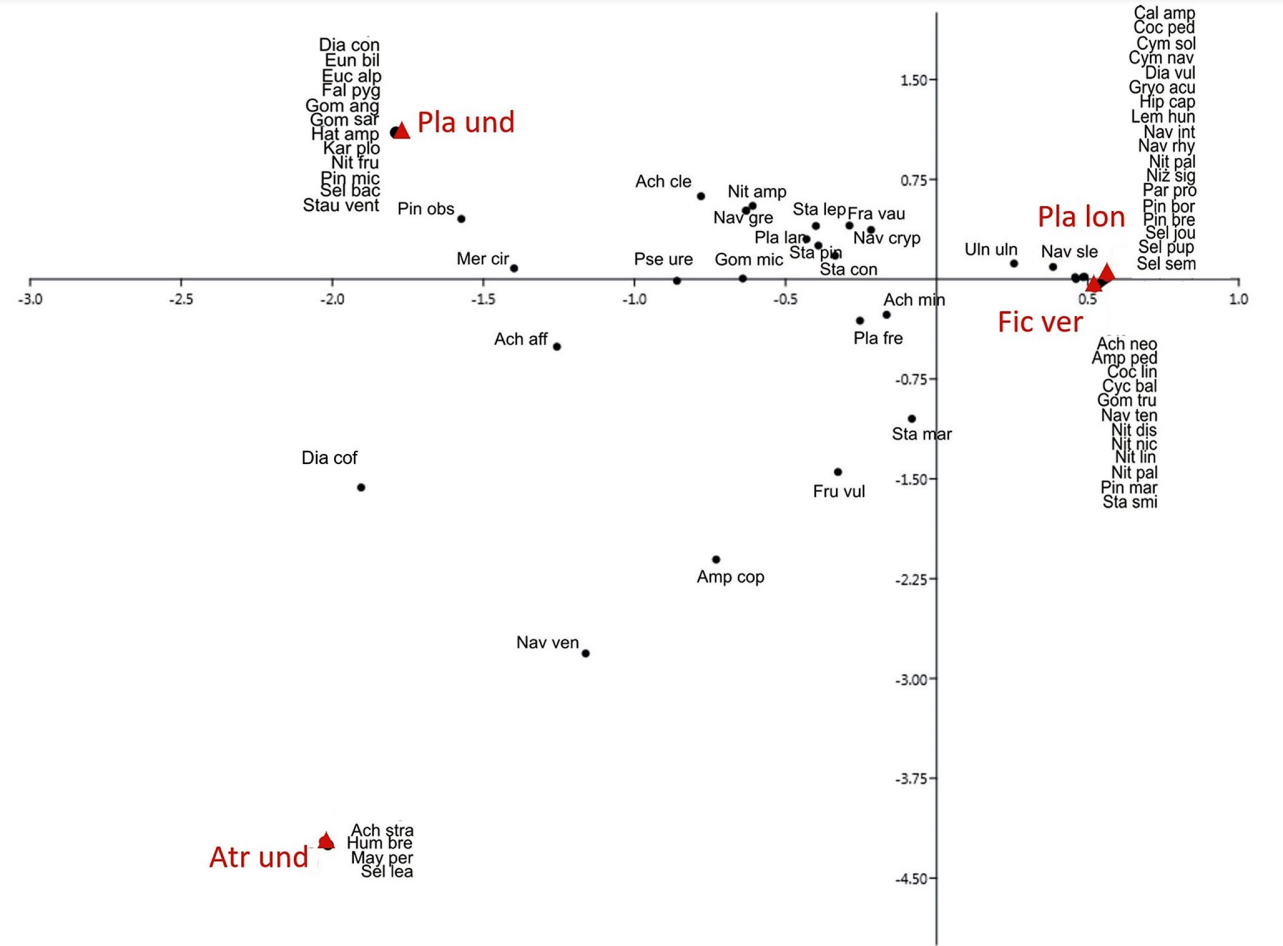
Correspondence analysis of diatoms growing on particular plant species. Pla lon – *Plagiothecium longisetum*; Fic ver – *Ficaria verna*; Pla und – *Plagiomnium undulatum*; Atr und – *Atrichum undulatum*, acronyms for green algae are in Table S1

**Fig. 6:**
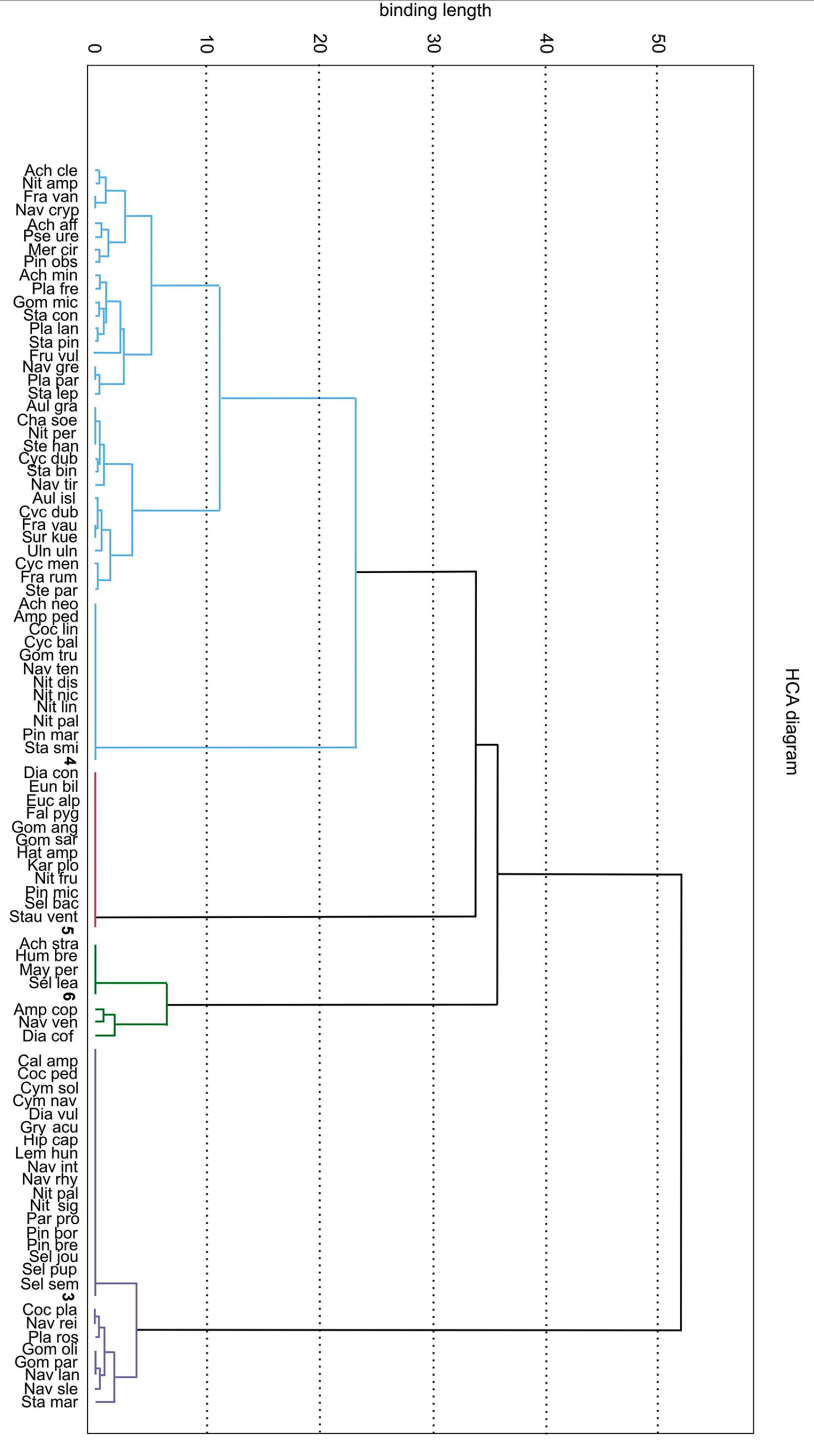
Dendrogram of similarity (Euclidean distance, Ward’s method) of plants and diatomaceous colonizers listed on them. Key: 3 – *Plagiothecium longisetum*; 4 – *Ficaria verna*; 5 – *Plagiomnium undulatum*; 6 – *Atrichum undulatum*, acronyms for diatoms are in Table S1

The CA showed a clear grouping of diatoms recorded on individual plants. Four clear groups were found to be associated with individual plants: *Plagiothecium longisetum*, *Ficaria verna*, *Plagiomnium undulatum* and *Atrichum undulatum* (Fig. 5). This observation is in line with the dendrogram of similarity (Fig. 6), where the analysis confirms the existence of the four groups described above. As the analyses show, certain diatoms tend to be host specific. The largest group of diatoms is associated with *Plagiothecium longisetum* (19 taxa), and the smallest with *Atrichum undulatum* (4 taxa), the latter group comprising only *Achnanthidium straubianum, Humidophila brekkaensis, Mayamea permitis* and *Sellaphora leavissima*.

### Plants as a substrate for cyanobacteria, green algae and diatoms

Most of the microalgae were recorded on terricolous host plants: *Polytrichum formosum* and *Dicranum polysetum* in the spruce monoculture, and *Plagiothecium longisetum*, *Ficaria verna* (vascular plant), *Plagiomnium undulatum* and *Articum undulatum* in the riparian forest. The fewest species (five taxa each) were recorded on *Polytrichum formosum* and *Dicranum polysetum*, and these were only green algae. The number of species and taxonomic diversity significantly increases in the riparian forest: the fewest taxa were found on *Atrichum undulatum* (24 taxa), with more (40 taxa) being found on *Plagiomnium undulatum* and *Ficaria verna* (50 taxa), and the most (65 taxa) recorded on *Plagiothecium longisetum* growing on the slope by the river.

Diatoms were the main colonizers of terricolous moss species in riparian area, i.e., *Achnanthidium minutissimum* (75 observations), *Staurosirella pinnata* (98 observations) and *Cyclostephanos* cf. *dubius* (120 observations), with these species being the most common on *Ficaria verna*. On the other hand, 21 diatom taxa were recorded least frequently on these substrates (e.g., *Caloneis amphisbaena*, *Cymbopleura naviculiformis*, *Eucconeis alpestris* and others), with their presence limited to one occurrence for each plant (Table S1).

One epiphyte was studied: the moss *Hypnum cupressiforme*, growing in the riparian forest on the bark of *Carpinus betulus* and *Acer pseudoplatanus*. However, not a single blue-green algae or diatom specimen was found on this species from either *Carpinus betulus* or *Acer pseudoplatanus.* Here the only colonizers were the green algae, but only a few taxa were recorded. Interestingly, similar numbers of taxa were recorded in both specimens: five on *H*. *cupressiforme* overgrown *Acer pseudoplatanus* and four on *H*. *cupressiforme* overgrown *Carpinus betulus*. Among the green algae, the most common were *Pseudochlorella signiensis* (42 records on *C. betulus* and 38 on *A. pseudoplatanus*) and *Pseudostichococcus* cf. *monallantoides* (22 and 33 records, respectively).

**Fig. 7:**
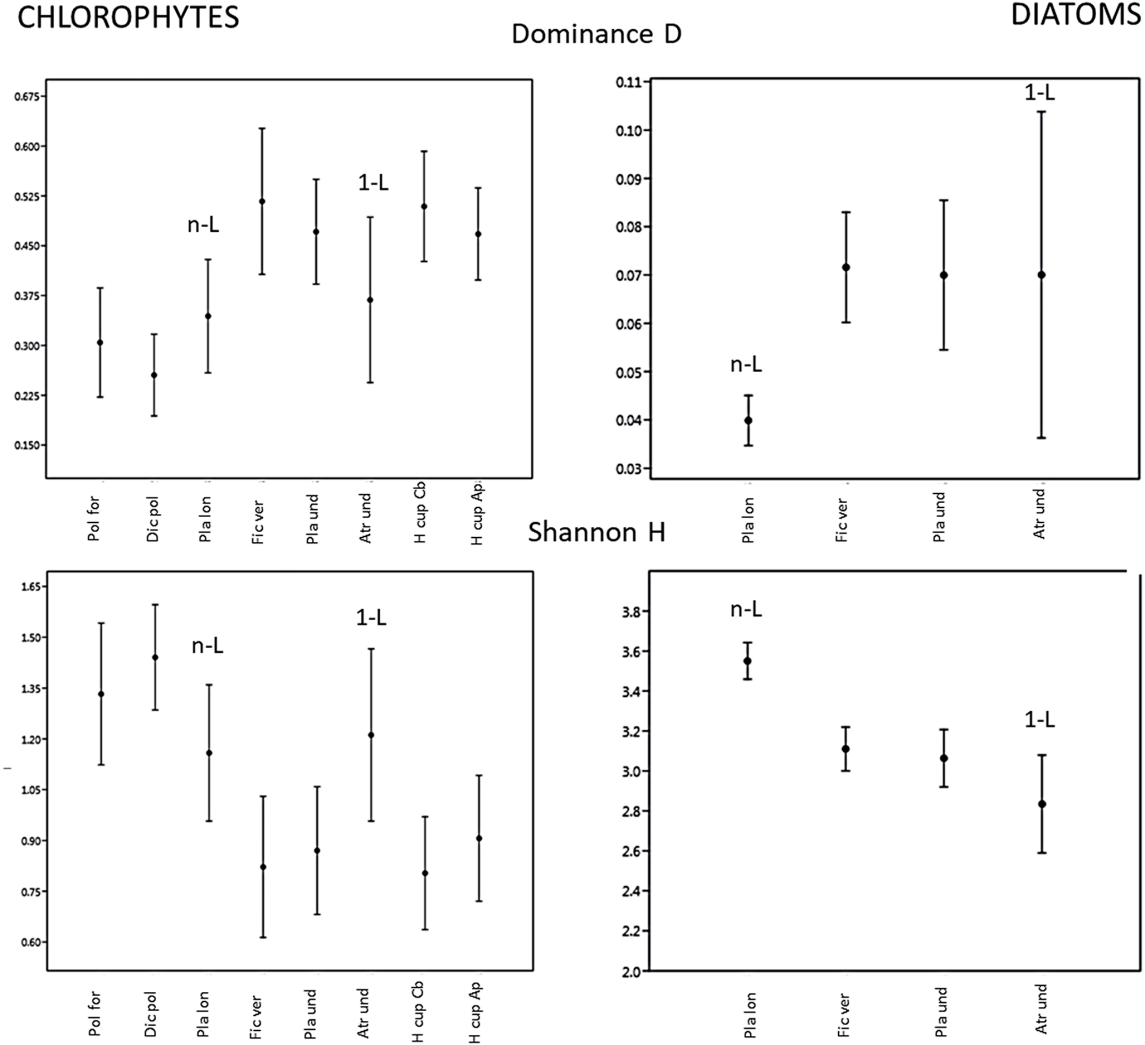
Alpha-diversity indices calculated for host plants regarding green algae (chlorophytes) and diatom communities; whiskers indicate confidence intervals and -L symbols refer to the morphology of leaves (n-L: leaf with many lamellae, 1-L: leaf with few lamellae, no symbol: leaf without lamellae); Pol for – *Polytrichum formosum*; Dic pol – *Dicranum polysetum*; Pla lon – *Plagiothecium longisetum*; Fic ver – *Ficaria verna*; Pla und – *Plagiomnium undulatum*; Atr und – *Atrichum undulatum*; Hyp cup Cb *– Hypnum cupressiforme* (on *Carpinus betulus*); Hyp cup Ap – *Hypnum cupressiforme* (on *Acer pseudoplatanus*)

The greatest diversity of green algae, and lowest species dominance, was recorded on mosses from the spruce monoculture area, while the lowest diversity, together with high dominance of few species, was noted on an epiphytic bryophyte in the riparian area (Fig. 7). In diatoms, the highest diversity and the lowest dominance of taxa was recorded in the community inhabiting *Plagiothecium longistetum*: a bryophyte growing on scarp next to the river; the diversity fell as the distance from the river increased, but with a similar Dominance D index. Moreover, no substantial changes in algal diversity appeared to be driven by leaf micromorphology (Fig. 7).

**Fig. 8:**
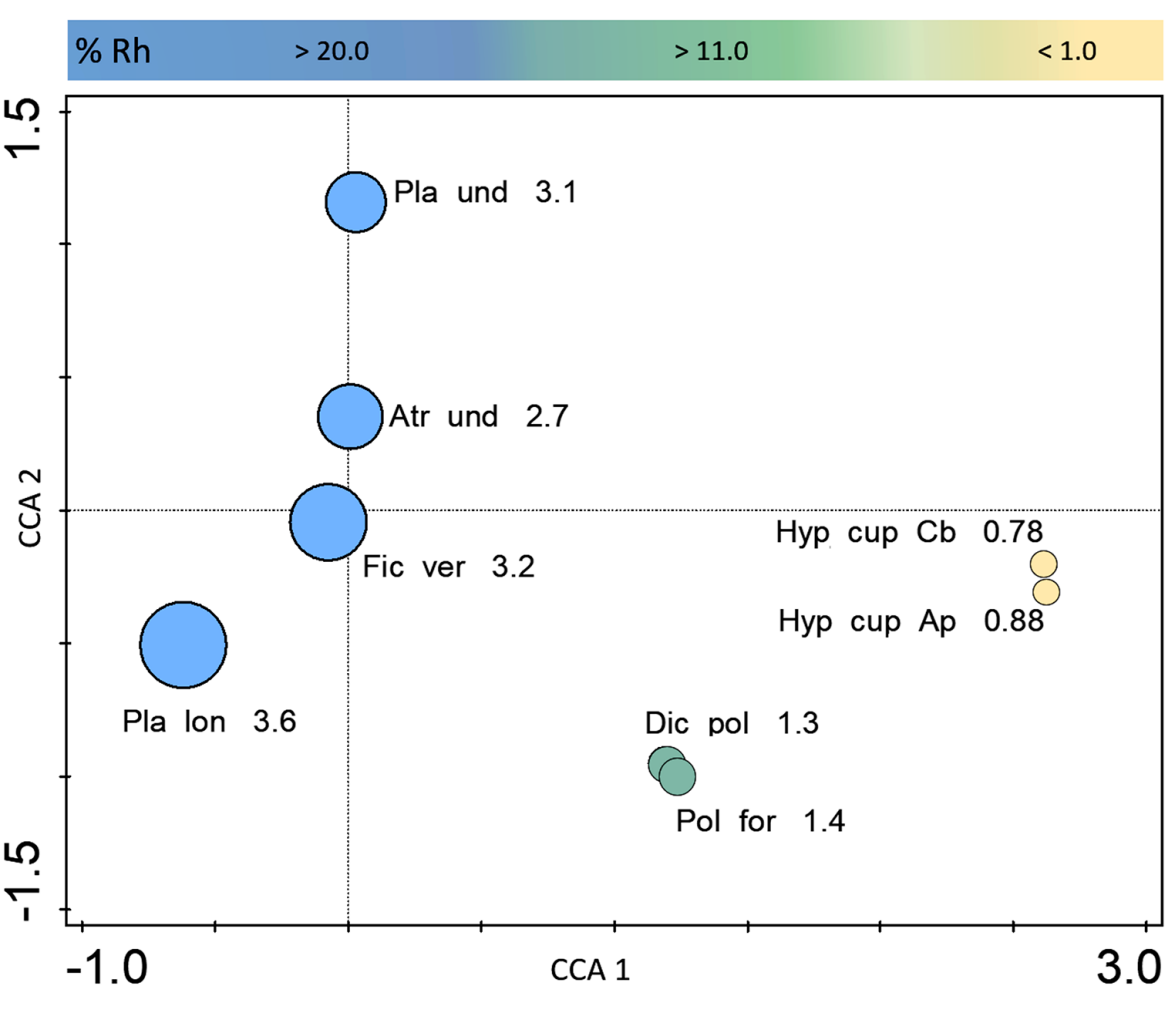
Distribution between plant hosts in ordination space regarding the variation in species composition (cyanobacteria, green algae and diatoms), explained by the relative humidity of substrate with Shannon-Weiner diversity index value. Key: Pol for – *Polytrichum formosum*; Dic pol – *Dicranum polysetum*; Pla lon – *Plagiothecium longisetum*; Fic ver – *Ficaria verna*; Pla und – *Plagiomnium undulatum*; Atr und – *Atrichum undulatum*; Hyp cup Cb *– Hypnum cupressiforme* (on *Carpinus betulus*); Hyp cup Ap – *Hypnum cupressiforme* (on *Acer pseudoplatanus*)

In multivariate data analysis, the relative humidity of substrates explained 89.57% of the total variation in the species composition. Both epiphytic bryophytes, from two different trees, as well as two terricolous species from spruce monoculture were grouped closely to each other, demonstrating similar algal communities with low taxonomic diversity (Fig. 8). The diversity of the algal communities increased three-fold as the humidity of the plant hosts increased, but the composition of algae differed between individual substrates: the analysis arranged bryophytes on different sides of the horizontal axis (*Plagiothecium longisetum* vs., *Atrichum undulatum* and *Plagiomnium undulatum*) (Fig. 8).

On all examined bryophytes, 87 taxa were recorded, including two cyanobacteria, seven green algae and 78 diatom taxa. Among the dominant taxa, the most frequently noted were *Pseudostaurosira brevistriata* (32 records), *Achnanthidium minutissimum*, *Cyclostephanos* cf. *dubius* (46 records) and *Staurosirella pinnata* (64 records). The rest of the species were much rarer, with some being found in isolated cases (Table S1).

The vascular plant (*Ficaria verna*) was relatively rich in algal species. In total, 50 taxa were recorded on *F. verna*, including one species of cyanobacteria, four green algae and 45 diatom taxa. Moreover, 12 diatom taxa (i.e., *Cyclotella balatonis, Nitzschia linearis, N. palea* and others) were found exclusively on a macrophyte clump; however, in low quantity (from 8 to 1 record). The predominant diatoms included *Staurosirella pinnata* (34 records) *Aulacoseira granulata* (45 records) and *Cyclostephanos* cf. *dubius* (74 records); the remaining species were much rarer, even sporadic, as indicated by the diatom communities presenting the highest Dominance D index (0.072) (Fig. 7, Table S8). Considering all algal groups, *F. verna* was the second greatest host in terms of algal diversity (Shannon-Wiener index – 3.2) (Fig. 8).

## Discussion

The algae form an ecologically and evolutionary diverse group of micro- to macroscopic photosynthetic organisms (Norton et al. 1996; Parker et al. 2008). The number of known algal species is estimated to vary from 150 000 to even one million taxa (Guiry 2012; Guiry and Guiry 2023), and most of these organisms can be found in aquatic ecosystems. However, water is not the only environment where algae can successfully develop. A small group of algae, including the cyanobacteria, green algae and diatoms, possesses the ability to survive and reproduce in terrestrial habitats. The presence of these phototrophs, often called aerophytic/terrestrial or airborne algae (i.e., algae free-floating in the atmosphere) was first reported in 1844, when Ehrenberg identified 18 species of diatoms that had come from Africa, together with the dust samples sent by Darwin (Sharma et al. 2007). Although some papers concerning terrestrial algae have since been published, this branch of phycological studies remains far less explored than that of the aquatic algae. Our findings indicate that among the microalgae found on the studied plants, the greatest taxonomic diversity was demonstrated by the diatoms (Bacillariophyta); in contrast, much fewer green algae taxa (Chlorophyta), and only three blue-green alga taxa (Cyanobacteria) were found in the algal material after cultivation.

The diatoms constitute the most numerous group of algae, with over 200,000 described taxa (Vanormelingen and Verleyen 2007), and they usually create communities of high taxonomic diversity. In ecosystems characterized by specific environmental conditions, i.e., a single cold-water spring, they can number over 200 taxa (Żelazna-Wieczorek 2011). In contrast, most cyanobacteria prefer humid environments with high temperatures. Their diversity in terrestrial environments increases towards the equator, but even on hard substrates in deserts, they may have a higher share than other photobiont groups (Büdel 1999; Cutler et al. 2013). Our field survey was performed in early spring, when atmospheric conditions do not favour the development of many cyanobacteria taxa in biofilms; however, the ecology of this group is very broad, and they have been identified from polar regions to the tropics and may even develop in extremely hot deserts (Dabravolski and Isayenkov 2022; Kvíderová et al. 2019). In the present study, they were not found in their vegetative stage in freshly-collected samples; however, they were observed in culture, suggesting they occurred on the studied plants in the form of dormant cells (akinetes), typical of heterocytous filamentous genera such as *Desmonostoc*, or in a metabolic resting stage (*Microcoleus, Gloeocapsa*) (Tashyreva and Elster 2012). It seems that the environmental and microhabitat conditions during the study period were not favourable or optimal for the growth of these three taxa.

The only group of algae that can be considered constant colonizers of the studied land plants was the chlorophytes: green algae were present on all moss turfs, as well as on the macrophyte clump. Regarding the alpha-diversity indices, the highest diversity of green algae was detected in spruce monoculture, where substrates had ca. 11% of Rh; however, surprisingly, it decreased in humid riparian area. It was anticipated that the least diversity would be observed in the case of *Hympnum coupressiforme* communities (< 1% Rh), which were characterised by very low humidity. In this case, two Trebouxiophyceae algae (*Pseudochlorella signiensis* and *Pseudostichococcus* cf. *monnalantoides*) dominated, as these algae can be found in almost all types of habitats from aquatic to terrestrial and can even develop in desert soil samples (Lewis and Lewis 2005; Pröschold and Darienko 2020). However, two plants, *Ficaria verna* and *Plagiomnium undulatum* (> 20%Rh), demonstrated equally low diversities of chlorophytes. In this case, the Shannon H index was also strongly shaped by two dominants: *Chlorella* sp. and *Klebsormidium flaccidum*; while both taxa are recorded in aquatic and terrestrial biofilms, *Klebsormidium* is generally better adapted to more hydrophilic habitats (Guiry and Guiry 2023; Mikhailyuk et al. 2015). Aerophytic green algae have been found to demonstrate a high tolerance to low humidity: they have developed many strategies to resist the effect of desiccation stress and some species are known to possess desiccation tolerance for up to 80 days (Aigner et al. 2020; Lüttge and Büdel 2010; Nowicka-Krawczyk et al. 2022; Terlova et al. 2021).

Diatoms were recorded only on the vascular plant and three moss species, *viz. Plagiothecium longisetum*, *Plagiomnium undulatum* and *Articum undulatum* in the riparian forest. These plants demonstrated the highest moisture level, ranging from 25.9 to 20.0% of Rh. Riparian forests are characterized by a very large variety of microhabitats, together with high soil moisture, the presence of aquatic ecosystems and a specific microclimate. All this contributes to high biodiversity (Wolski 2013; Żarnowiec 1995). Among the environmental factors known to influence the structure of terrestrial diatom communities, key roles are believed to be played by pH, organic matter content and moisture (Foets et al. 2020, 2021; Stanek-Tarkowska et al. 2018), with the latter being the limiting factor for diatom development (Antonelli et al. 2017; Chen et al. 2012); therefore, none were observed on substrates with a moisture content below 20% Rh. The dry coniferous forest, associated with a low groundwater level and a predominance of sandy soils, is usually considered quite poor in terms of algal biodiversity (Neustupa and Škaloud 2010; Quin et al. 2016; Štifterová and Neustupa 2015). No diatoms were present in the studied communities, and only a few taxa of green algae were found.

The diversity in diatom communities in the riparian forest decreased with the distance of the plant from the river: the highest was recorded on moss turf growing on scarp (*P. longisetum*), and the lowest on moss from wet soil in a distance of six meters from the river (*A. undulatum*). Regarding the qualitative and quantitative structure of communities and the autecology of taxa, a high share of freshwater diatoms was noted on plants growing closest to the river such as, *inter alia*, *Amphora copulata, Achnanthidium minutissimum, Cocconeis pediculus, C. placentula, Cymatopleura solea, Gomphonema olivaceum, G. parvulum, Navicula lanceolata, N. slesvicensis* and *Staurosirella pinnata*.

The CA found considerable similarity between the diatom communities of *F. verna* and *P. longisetum*; however, while the macrophyte *F. verna* is periodically flooded by water, and may hence be colonised by various freshwater taxa, *P. longisetum* grows above the river and it does not have any direct connection to water. Nevertheless, some reports suggest that freshwater diatoms could transfer from water to other habitats via airborne water vapour (Foets et al. 2020, 2021; Hetko et al. 2022; Stanek-Tarkowska et al. 2018). Alternatively, diatom cells may be carried to moss turf during rainfall by river water being splashed on the scarp by raindrops.

Regarding the autecology of taxa, our analysis based on the Van Dam et al. ecological descriptor confirmed the presence of several strictly aerophilic species in all diatom communities: *Pinnularia borealis* and *P. brebissonii* on *Plagiothecium longisetum*; *Navicula tenelloides* on *Ficaria verna*; *Humidophila contenta* and *Hantzschia amphioxys* on *Plagiomnium undulatum*; *Humidophila brekkaensis* on *Atrichum undulatum*. The analysis was performed in Omnidia 6.1 software, which possesses a very reliable database of around 28,000 diatom taxa together with the ecological classification of taxa. Van Dam et al. (1994) classify aerophilic taxa as those mainly occurring in wet and moist or temporarily dry places. This ecological classification also includes a *terrestrial* category for species nearly exclusively occurring outside water bodies. No terrestrial species were found in this study; therefore, the diatom communities colonizing studied plants can be considered as associations from the border between the aquatic and aerophytic environments.

The epiphytic diatoms have developed specific adaptations to attach to the plant substrate, such as the production of mucopolysaccharide stalks or the secretion of sticky mucopeptide substances (Letáková et al. 2018; Riato and Leira 2020; Rojas and Hassan 2017). Among the identified diatoms, *Achnanthidium minutissimum*, *Cocconeis placentula*, *C. lineata*, *C. pediculus*, *Gomphonema olivaceum*, *G. parvulum*, *Fragilaria vaucheriae*, prefer an epiphytic lifestyle and can quickly attach to the plant substrate.

While the gametophyte of mosses is usually a leafy stem, the moss leaves themselves demonstrate a range of shapes, dimensions and structures, as well as micromorphology types. Some taxa have a smooth leaf surface (e.g., species of *Plagiothecium*), others possess various numbers of lamellae on the ventral side characteristic of a particular taxon: the genus *Atrichum* possesses a few, while the genus *Polytrichum* can possess several dozen (Nyholm 1965; Smith 2001). One aim of the present study was to confirm whether the micromorphology of moss leaves affects the number of algae colonizing them. However, our findings indicate that while the number of recorded algae is influenced by the humidity of the substrate, micromorphology does not appear to play a role. For instance, the least number of algal taxa (*n* = 5) was demonstrated by *Polytrichum formosum*, characterized by the richest micromorphology, which grew over the driest habitat; in contrast, the richest range of species (*n* = 64) was associated with *Plagiothecium longisetum*, a smooth-leafed moss growing on the escarpment in the riparian forest. The micromorphology of moss leaves had no significance in the colonization of epiphytic diatoms.

Similarly, taking into consideration the time of substrate availability, the degree of microalgae colonization did not appear to significantly differ between the `periodic` plant (*Ficaria verna*) and mosses growing throughout the year. *F. verna* demonstrated moderate diversity of diatoms; its green alga community was less rich, but with roughly the same diversity as in *Plagiomnium undulatum* or *Hynum cupressiforme*. The degree of colonization is clearly influenced by the time that the substrate is exposed for potential inhabitants (Nowicka-Krawczyk et al. 2022). In this case, the high diversity of microalgae on *F. verna*, indicated by the Shannon-Weiner index for the total algal community, was strongly impacted by diatom inhabitants which were introduced by water from the river. Nevertheless, although a macrophyte may only exist for a short while, it can still be colonised by green algae: they can be carried onto the vascular plant anytime by the wind.

## Conclusions

Our findings show that even such specific microhabitats as terrestrial bryophytes and higher plants in the temperate zone can harbour a high taxonomic diversity of microalgae. Such high biodiversity is also influenced by the diversity of abiotic parameters found in riparian forests. However, in the case of moss on tree bark, the limiting factor for algal colonization appears to be the humidity level. Indeed, samples from dry coniferous forest, associated with a low groundwater level and a predominance of sandy soils, also demonstrated low algal species diversity.

The micromorphology of moss leaves has no clear influence on the degree of algal colonization. Although a higher number of lamellae on the leaf is associated with greater micro-unevenness, providing a good spot for cell adhesion, the diversity of algae depends rather on the total humidity of turfs than on the shape of moss leaves.

It can also be tentatively concluded that the length of vegetation of the substrate does not have a significant influence on the species diversity of its microalgae; indeed, the two plants with the greatest numbers of identified species had different vegetation periods: *Plagiothecium longisetum*, a bryophyte with year-round vegetation, and *Ficaria verna*, a higher plant with a short vegetational period. Hence, it is not the durability of the substrate throughout the year that limits the occurrence of algae, but rather the availability of water and potential impact of aquatic communities.

## Electronic supplementary material

Below is the link to the electronic supplementary material.

Supplementary Material 1

## Data Availability

All data generated or analysed during this study are included in this published article [and its supplementary information files]. Both the plants and the area from which they were collected are not protected by law. The specimens studied are common and very common taxa throughout Europe or even the entire Northern Hemisphere, not covered by any legal protection. Thus, all methods were performed in accordance with the relevant guidelines/regulations/legislation national law.
